# Evaluating prevalence of depression, anxiety and hopelessness in patients with Vitiligo on an Iranian population

**DOI:** 10.1186/s12955-020-1278-7

**Published:** 2020-02-03

**Authors:** Nasrin Hamidizadeh, Sara Ranjbar, Ahmad Ghanizadeh, Mohammad Mahdi Parvizi, Peyman Jafari, Farhad Handjani

**Affiliations:** 1 Molecular Dermatology Research Center, Shiraz University of Medical Sciences, P.O. Box: 7134844119, Zand Avenue, Shiraz, Iran; 2 Research Center for Psychiatry and Behavioral Sciences, Department of Psychiatry, Shiraz University of Medical Sciences, Shiraz, Iran; 3 Department of Psychiatry, UCLA-Kern Psychiatry Residency Program, Kern Medical, Kern Behavioral Health and Recovery Services, Bakersfield, CA USA; 4 Department of Biostatistics, Shiraz University of Medical Sciences, Shiraz, Iran

**Keywords:** Vitiligo, Depression, Anxiety, Hopelessness and general health

## Abstract

**Introduction:**

Vitiligo is caused by partial or complete destruction of melanocytes in the affected skin area and influences the patient’s quality of life. Besides physical involvement, vitiligo patients experience a high level of stress. Depression and Anxiety are common psychiatric disorders in vitiligo patients.

**Aim:**

This study, as the first study, evaluates hopelessness, anxiety, depression and general health of vitiligo patients in comparison with normal controls in an Iranian population.

**Method:**

Hundred patients with vitiligo and hundred healthy controls were examined. General health, depression, hopelessness and anxiety were evaluated based on general health questionnaire. Anxiety, depression and hopelessness levels were analyzed using Chi–Square, and the mean value of general health was evaluated through t-test.

**Results:**

The results showed that anxiety and hopelessness levels were significantly higher in vitiligo patients than those who are in healthy controls. This significant difference refers to high levels of anxiety and hopelessness among women with vitiligo.

It was also found that the single patients were more anxious, hopeless and depressive, while the married patients were only more anxious and hopeless than those who are in the control group, respectively.

General health of patients was significantly worse than in healthy controls. The low level of general health in patients was related to poorer level of general health among women with vitiligo.

**Conclusion:**

It seems that women with vitiligo are more mentally stressed than men with vitiligo. Both singles and married vitiligo patients suffer from anxiety and hopelessness.

## Introduction

Vitiligo is a chronic systemic disease which is characterized by hypopigmented macules and is caused by partial or complete destruction of melanocytes in the affected skin [1–3]. However, the exact cause of vitiligo is unknown; evidence suggests that various factors such as autoimmune, genetic and environmental factors are involved in the development of this disease. It affects 0.5–2% of the population worldwide regardless of race and gender [4, 5]. Women and men are equally affected by the vitiligo [6]. Although it can initiate at any time [7, 8], the initiation in 50% of the people is before 20 years [9]. Depending on how soon it starts, more skin will be damaged. Low prevalence of vitiligo is observed in Scandinavian countries while Asian, especially Indians and Middle Eastern are at greater risk [10]. It should be noted that vitiligo is usually asymptomatic; it does not shorten the patient’s life time and does not reduce physical activity [11, 12]. However, it results in several limitations such as regular visits for PUVA/narrow band UVB therapy, immunosuppressive therapies and risk of carcinogenesis with phototherapy.

Although vitiligo is not contagious, its effect on quality of life is related to psychological problems such as low self-esteem, embarrassment, negative effect on sexual relations, social isolation and experiencing vitiligo-related discrimination [13–15]. Therefore, it becomes a barrier for seeking job and marriage; and social stigma and suicidal ideation have been also reported [16].

Vitiligo patients experience a high level of stress and psychiatric disorders in addition to physical involvement. Depression, anxiety, suicidal thoughts, suicidal attempts, embarrassment, social problems, discomfort, cognitive impairment, embarrassment, and physical limitation were reported in vitiligo patients [17, 18]. For this reason, vitiligo influences the patients’ quality of life; so, related psychiatric disabilities should not be underestimated [19].

Furthermore, the prevalence of depression has increased in Europe, Asia, Africa, and the Middle East in the recent years [20].

The present study evaluated hopelessness, anxiety, depression and general health of vitiligo patients who were attended in the phototherapy center at Faghihi Hospital. Meanwhile, considering the fact that vitiligo causes and related psychological disorders vary in different societies; psychological problems of patients with vitiligo were investigated in Shiraz.

For this purpose, general health, depression, hopelessness and patients’ anxiety were evaluated based on General Health Questionnaire-28 (GHQ-28) [21], Beck’s Depression Inventory [22], Beck Hopelessness Scale [23] and Beck Anxiety Inventory [24], respectively.

## Method

This study was conducted at Molecular Dermatology Research Center which is affiliated to Shiraz University of Medical Sciences and Dermatology Clinic of Faghihi Hospital in Shiraz during one and a half year.

Vitiligo patients, who referred dermatology clinic and were being treated with phototherapy, were randomly allocated to case and control group based on black permutation design.

All patients of both genders, who were at least 18 years old, without any serious mental and physical disability, and those who consented to participate were enrolled in this study [17]. The sample size was estimated based on the mean score (standard deviation) of Beck’s depression inventory. The sample sizes was calculated according to previous studies between vitiligo patients [25] and healthy subjects (control group), respectively [26, 27]. Finally, 100 patients and 100 healthy controls were enrolled in the study. Based on error rate of type I and according to formula: *N* = 4 $$ {\sigma}^2{\left(z\frac{a}{2}+ z\beta \right)}^2/{\left(\mu -{\mu}_2\right)}^2 $$, α = 0.05, β = 0.2, power (1-β) = 80%, $$ \raisebox{1ex}{${\mu}_{1-}{\mu}_2$}\!\left/ \!\raisebox{-1ex}{$\sigma $}\right.=\raisebox{1ex}{$1$}\!\left/ \!\raisebox{-1ex}{$2.5$}\right.=0.4. $$

Data were collected, using Beck’s Depression Inventory (BDI), Beck Hopelessness Scale (BHS), Beck Anxiety Inventory (BAI), and General Health Questionnaire (GHQ 28) after consulting with a psychology professor.

Validity and reliability of these questionnaires were evaluated in the relevant studies [28–30]. Written informed consent was obtained from each participant and then, patient’s information forms and the questionnaires were completed by each patient after the disease confirmation by a dermatologist.

Beck’s Depression Inventory includes 21 questions, which evaluates the feelings of sadness, guilt, lack of interest, social isolation and suicidal ideation. Beck Hopelessness scale contains 20 statements, which measures negative attitudes or pessimism about the future, life prospect, achieving his/her desires or trusting the future.

Beck’s Anxiety Inventory contains 21 questions which measures anxiety, difficulty relaxing, nervous tension, agitation and restlessness during the past week. General health was also measured through a General Health Questionnaire including 21 questions, which asks respondents to report how they felt during the last 4 weeks on a range of symptoms including somatic symptoms, anxiety, insomnia, social dysfunction and severe depression. Patient demographic characteristics such as age, age of onset, gender, hereditary disease, comorbidities, disease-related involvement of the body surface, type of treatment and marital status were added to the questionnaires.

Statistical analysis was done using SPSS statistical software, version 18 (IBM, Armonk, NY, USA). The levels of anxiety, depression and hopelessness were measured using Chi–Square; the mean value of general health was evaluated through *t*-test and correlation coefficient was calculated using Spearman’s rank correlation coefficient. In all analytical tests, *p* value more than 0.5 was considered significant.

## Results

Generally, 100 vitiligo patients and 100 healthy controls (as the control group) participated in this study, among which 134 were female and 66 male. The mean age of the patients and control group was 34.50 ± 12.225 and 37.300 ± 10.209 years, respectively, which was not significantly different (Table 1).

**Table 1:** Shows demographic characteristics of vitiligo and control group

	*n*	Age (Mean ± SD)	*p* – value
Case	100	34.50 ± 12.225	0.081
Control	100	37.30 ± 10.209	
Sex			
Case			
Female	69	34.22 ± 11.697	0.732
Male	31	35.13 ± 13.515	
Control			
Female	65	36.23 ± 9.548	0.160
Male	35	39.26 ± 11.200	
Case			
Age of onset of vitiligo			
Female	69	22.94 ± 14.396	0.373
Male	31	25.61 ± 12.339	
Duration of disease			
Female	69	11.28 ± 10.222	0.425
Male	31	9.52 ± 10.013	
Marital status (missing = 5)			
Married			
Case	56	39.02 ± 11.298	0.565
Control	53	40.13 ± 8.614	
Single			
Case	42	28.10 ± 10.164	0.075
Control	39	32.03 ± 9.413	
Widow			
Case	1	59	0.445
Control	4	50.75 ± 8.421	
Family history of vitiligo			
Female	22	32.50 ± 11.300	0.445
Male	7	36.71 ± 16.070	

The results showed that all three variables (hopelessness, anxiety and depression) had a positive and significant relationship with the disease duration. Age had negative relationship with hopelessness, anxiety and depression. It was significantly related to levels of hopelessness and depression, but it had not significant relationship with the level of anxiety (Table 2).

**Table 2:** Shows the correlation between depression, anxiety and hopeless levels with age A: in case group and B: in control group, and C: shows correlation between depression, anxiety and hopeless levels with duration of disease in vitiligo group

	Correlation Coefficient	*p-valu*e
A: Case (*n* = 100)		
Age		
Depression	- 0.255	0.010
Anxiety	- 0.147	0.150
Hopeless	- 0.243	0.015
B: Control (n = 100)		
Age		
Depression	0.051	0.619
Anxiety	0.026	0.801
Hopeless	- 0.011	0.911
C: Case (n = 100)		
Duration of illness		
Depression	0.194	0.053
Anxiety	0.293	0.003
Hopeless	0.216	0.031

The levels of hopelessness, anxiety and depression were also measured in both patient and healthy controls based on gender. The results also showed that the patients were significantly more anxious and hopeless compared to the healthy controls while there was no difference in the level of depression between the patients and healthy controls. On the other hand, the women with vitiligo were significantly more anxious and hopeless than men with vitiligo (Tables 3 and 4).

**Table 3:** Show the prevalence and severity of depression, anxiety and hopelessness in the case and control group

	Case	Control	*p-value*
Severity of depression			
Minimal	53 (53.0%)	60 (60%)	0.157
Mild	19 (19.0%)	25 (25%)	
Moderate	16 (16.0%)	9 (9%)	
Severe	12 (12.0%)	6 (6%)	
Severity of anxiety			
Minimal	32 (32. 7%)	49 (49.5%)	0.015
Mild	27 (27.6%)	30 (30.3%)	
Moderate	25 (25.5%)	15 (15.2%)	
Severe	14 (14.3%)	5 (5.1%)	
Severity of hopelessness			
Minimal	40 (40.0%)	48 (48.5%)	0.006
Mild	34 (34%)	42 (42.4%)	
Moderate	18 (18.0%)	9 (9.1%)	
Severe	8 (8.0%)	0 (0%)

**Table 4:** Shows the prevalence and severity of depression, anxiety and hopelessness in women and men in the case and control group

	Women			Men		
	Case	Control	*p-value*	Case	Control	*p-value*
Severity of depression						
Minimal	36 (52.2%)	39 (60.0%)	0.203	17 (54.8%)	21 (60.0%)	0.698
Mild	12 (17.4%)	16 (24.6%)		7 (22.6%)	9 (25.7%)	
Moderate	12 (17.4%)	5 (7.7%)		4 (12.9%)	4 (11.4%)	
Severe	9 (13.0%)	5 (7.7%)		3 (9.7%)	1 (2.9%)	
Severity of anxiety						
Minimal	15 (22.4%)	34 (53.1%)	0.002	17 (54.8%)	15 (42.9%)	0.354
Mild	21 (31.3%)	17 (26.6%)		6 (19.4%)	13 (37.1%)	
Moderate	21 (31.3%)	10 (15.6%)		4 (12.9%)	5 (14.3%)	
Severe	10 (14.9%)	3 (4.7%)		4 (12.9%)	2 (5.7%)	
Severity of hopelessness						
Minimal	26 (37.7%)	36 (56.3%)	0.021	14 (45.2%)	12 (34.3%)	0.093
Mild	24 (34.8%)	22 (34.4%)		10 (32.3%)	20 (57.1%)	
Moderate	14 (20.3%)	6 (9.4%)		4 (12.9%)	3 (8.6%)	
Severe	5 (7.2%)	0 (0.0%)		3 (9.7%)	0 (0.0%)	

Married patients were more anxious and hopeless based on their levels of hopelessness, anxiety and depression in comparison with the healthy control. The results of this study also showed that depression, anxiety and hopelessness levels of single patients were higher than the single subjects in the healthy controls. There was no significant difference in the levels of depression, anxiety and hopelessness between married and single vitiligo patients (Table 5).

**Table 5:** Shows the prevalence and severity of depression, anxiety and hopelessness in married and single patients in the case and control group

	Married			Single		
	Case	Control	*p-value*	Case	Control	*p-value*
Severity of depression						
Minimal	35 (62.5%)	43 (81.1%)	0.137*	17 (40.5%)	14 (35.9%)	0.033*
Mild	8 (14.3%)	2 (3.8%)		10 (23.8%)	20 (51.3%)	
Moderate	8 (14.3%)	5 (9.4%)		8 (19.0%)	2 (5.1%)	
Severe	5 (8.9%)	3 (5.7%)		7 (16.7%)	3 (7.7%)	
Severity of anxiety						
Minimal	21 (38.9%)	33 (62.3%)	0.017*	10 (23.8%)	12 (31.6%)	0.008*
Mild	15 (27.8%)	7 (13.2%)		12 (28.6%)	21 (55.3%)	
Moderate	9 (16.7%)	11 (20.8%)		15 (35.7%)	3 (7.9%)	
Severe	9 (16.7%)	2 (3.8%)		5 (11.9%)	2 (5.3%)	
Severity of hopelessness						
Minimal	24 (42.9%)	34 (65.4%)	0.039*	15 (35.7%)	10 (25.6%)	0.040*
Mild	18 (32.1%)	14 (26.9%)		15 (35.7%)	24 (61.5%)	
Moderate	11 (19.6%)	4 (7.7%)		7 (16.7%)	5 (12.8%)	
Severe	3 (5.4%)	0 (0.0%)		5 (11.9%)	0 (0.0%)	

Regarding to the vitiligo-related involvement of the body surface, women whose hands were affected, and married patients with facial, arm, and hand involvement were significantly more anxious. Also, married patients whose genital areas were involved and single patients whose head/neck and trunk areas were involved were significantly more depressed (data was not shown**).**

Comparing the results of general health in patients with those of the control group showed that patients had significantly poorer general health than the healthy controls. Also, the results showed that women with vitiligo had poorer general health compared to women in the control group. However, the general health of men who suffer from vitiligo was not significantly different compared to the healthy men. There was no significant difference between women and men in relation to general health in patients and in controls, respectively (Table 6). Also, vitiligo involves body area which did not affect general health status (data not shown).

**Table 6:** shows GHQ positive and GHQ negative in the vitiligo group in comparison with the controls

	n	GHQ^a^(Mean ± SD)	*p*-value	GHQ -^b^ n (%)	GHQ + ^c^ n (%)	*p*-value
Case	99	26.46 ± 15.183	0.036	50 (50.5)	49 (49.5)	0.018
Control	100	22.12 ± 13.852		67 (67.0)	33 (33.0)	
Case						
Women	68	27.74 ± 15.554	0.219	33 (48.5)	35 (51.5)	0.666
Men	31	23.68 ± 14.181		17 (54.8)	14 (45.2)	
Control						
Woman	65	20.35 ± 13.008	0.082	45 (69.2)	20 (30.8)	0.656
Men	35	25.40 ± 14.939		22 (62.9)	13 (37.1)	
Women						
Case	68	27.74 ± 15.554	0.004	33 (42.3)	35 (63.6)	0.022
Control	65	20.35 ± 13.008		45 (57.7)	20 (36.4)	
Men						
Case	31	23.68 ± 14.181	0.634	17 (43.6)	14 (51.9)	0.618
Control	35	25.40 ± 14.939		22 (56.4)	13 (48.1)	

^a^General Health Questionnaire (GHQ)

^b^having normal mental health

^c^having poor mental health

## Discussion

Vitiligo is an acquired disease, which is caused by the loss of functioning melanocytes, and its cause is still unknown. There are different treatment options based on different mechanisms to treat vitiligo.

Although vitiligo does not cause physical disability, long-term treatment, lack of consistent effective therapy and a significant financial burden are stressful for patients who have been involved. This issue affects the patients’ emotions, mental well-being and sexual relationship, and has a significant effect on patients’ quality of life. In reviewing DLQI studies, patients’ quality of life with vitiligo has been evaluated over the past 20 years [31]. In several studies, poor quality of life of patients was observed in some countries [32] as well as in Iran [33–35]. Studies showed poorer quality of life in women with vitiligo compared to men with vitiligo [36], in married women in comparison with single women [33], and in Muslim women in comparison with Muslim men [37]. Nonetheless, depression [20, 25, 38, 39], anxiety and hopelessness [40, 41], were more reported in vitiligo patients.

In this study we investigated the hopelessness, depression, anxiety and general health levels of vitiligo patients. We found that the levels of anxiety and hopelessness in patients were significantly higher than healthy controls.

The results also showed that women with vitiligo compered to healthy controls were more anxious and hopeless while, there was no significant difference in the levels of anxiety and hopelessness among men with vitiligo in comparison with the healthy controls.

It seems that the significant difference in the levels of hopelessness and anxiety between the patients and controls was due to low significant levels of these two variables between women. Our results were consistent with the results of previous studies, which suggest women with vitiligo had poorer quality of life [33, 36, 37, 42]. However, other studies concluded that vitiligo patients suffer more from depression and anxiety [20, 38–41]. On the contrary to other studies, this study showed that the women were more anxious and hopeless. The married patients with genital areas and feet involvement and the single patients with trunk involvement were significantly more depressed.

The results also showed that there was a significant difference in general health between the patients, so that the patients had a worse general health. The significant difference in the general health between the patients and the healthy controls is due to a poorer general health of women with vitiligo. The results of this study also showed that married people and singles were more hopeless and anxious than their corresponding controls, whereby the singles were also even more depressed.

Some studies showed that the quality of life of vitiligo patients was affected by stigma [43], sexual dissatisfaction [44] and lack of self-esteem [45], and patients have difficulty to find a job and consider vitiligo as a significant obstacle to get married [16]. Therefore, control or treatment of vitiligo patients requires general identification of the patient’s problems, both psychological and physical, and individual therapy. In this sense, dermatologists should evaluate the psychiatric status of women with vitiligo in addition to physical treatment. Furthermore, the interaction between the patient and the doctor is also significant. Previous studies showed the effects of psychiatric disorders, patient-physician interaction, and also evaluation of patients’ mental and physical health on their quality of life [17, 46, 47]. Papadopoulos et al. showed that counseling can help to improve self-esteem and quality of life in vitiligo patients and may even have a positive effect on the course of the disease [48]. Other studies have confirmed that it is important to identify and treat patients’ psychosocial and social factors for more positive effect on quality of life and treatment [49, 50].

## Conclusion

Women suffer from vitiligo more than men and are more anxious and hopeless. The physician should consider both the physical and psychological problems especially in women in order to obtain the best possible treatment.

## Data Availability

The data is presented and kept by the authors and is available for scrutiny.

## Abbreviations

BAI: Beck Anxiety Inventory
BDI: Beck’s Depression Inventory
BHS: Beck Hopelessness Scale
DLQI: Dermatology Life Quality Index
GHQ: General Health Questionnaire
IBM: International Business Machines
NY: New York
SD: Standard deviation
USA: United States of America
